# Supplementary material for: Learning representations of chromatin contacts using a recurrent neural network identifies genomic drivers of conformation
Source: Nat Commun. 2022 Jun 28;13:3704. doi: 10.1038/s41467-022-31337-w (PMC9240038; doi:10.1038/s41467-022-31337-w)
Supplement: Supplementary file 1 — Supplementary Information [file 41467_2022_31337_MOESM1_ESM.pdf]

Learning representations of chromatin contacts using a recurrent neural network identifies genomic drivers of conformation: Supplementary Results

- Fig. 1 displays the R2 performance in HFF-hTERT and WTC11.
- Fig. 2 talks about the CTCF interaction dots.
- Fig. 3 displays the confusion matrix for the subcompartment classification task.
- Fig. 4 shows additional classification metrics for GM12878.
- Fig. 5 shows additional classification metrics for H1-hESC.
- Fig. 6 shows classification metrics for HFF-hTERT.
- Fig. 7 shows the feature importance scores for domain labels given by Segway-GBR.
- Fig. 8 shows possible ways to perform knockout at a given site.
- Fig. 9 gives an overview of CTCF and cohesin arrangement near domain edges.
- Fig. 10 displays the observed and predicted Hi-C values after TAL1 and LMO2 anchor deletions.
- Fig. 11 displays the prediction heatmaps for models trained on single-cell Hi-C (scHi-C).
- Fig. 12 shows the embedding neural network layer.
- Fig. 13 shows the ablation experiments carried out to determine the representation size of Hi-C-LSTM.
- Fig. 14 points out the salient features of Hi-C-LSTM predictions.
- Fig. 15 shows the results from the parameter search for the XGBoost classifier.
- Fig. 16 shows results from other ways to perform knockout like using the padding and average representations.
- Table. 1 shows the p-values for feature attribution scores from various elements.
- Table. 2 shows the links to datasets used in the study.
- Table. 3 shows the time taken to train and test all methods.

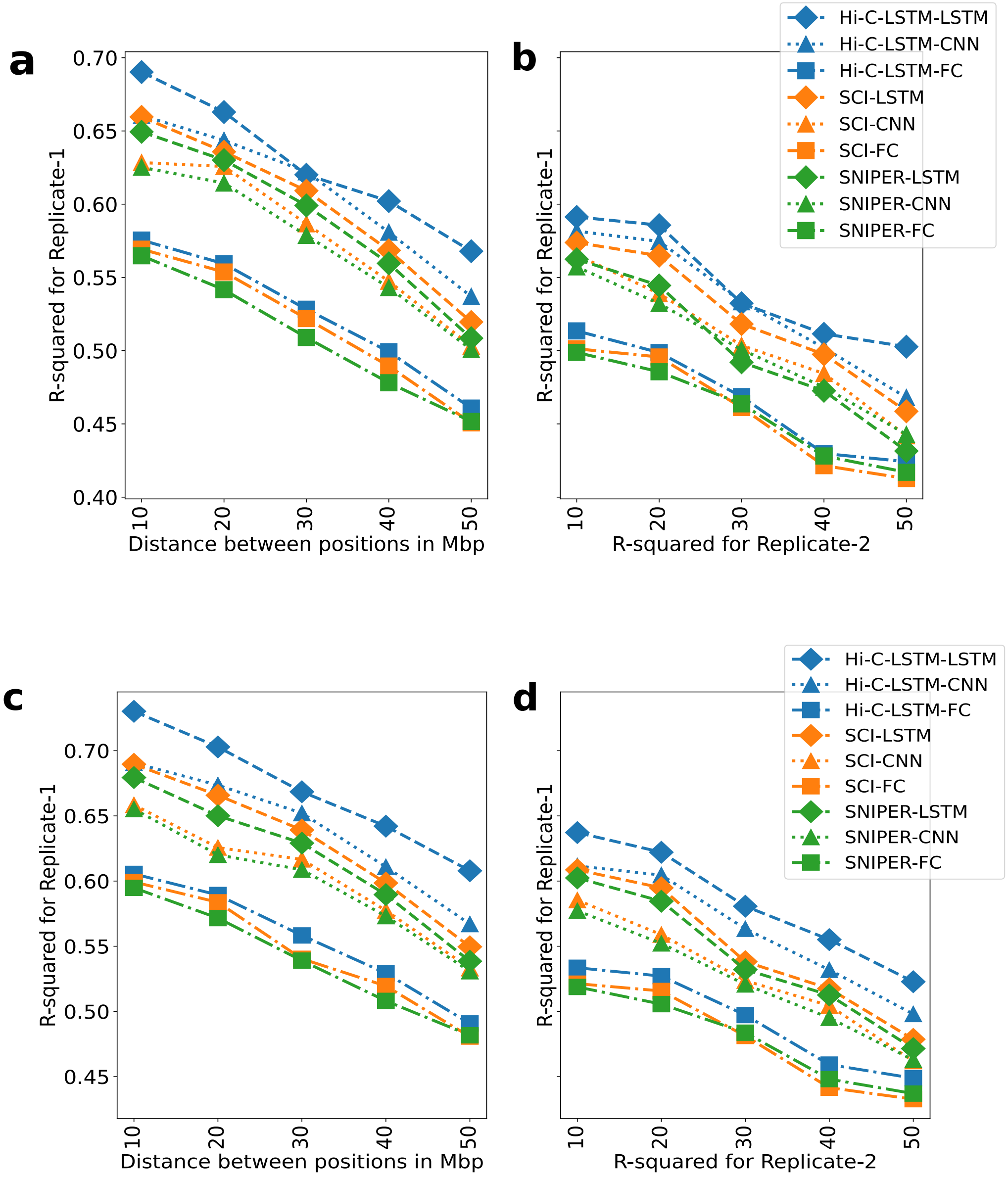

Figure 1: **Accuracy with which representations reproduce the original Hi-C matrix. a,b)** The Hi-C R-squared computed using the combinations of representations from different methods and selected decoders for replicate 1 and 2 (HFF-hTERT). The horizontal axis represents the distance between positions in Mbp. The vertical axis shows the average R-squared for the predicted Hi-C data. The R-squared was computed on a test set of chromosomes using selected decoders with the representations trained all chromosomes as input. The legend shows the different combinations of methods and decoders, read as *[representation]-[decoder]*. **c,d)** Same as a,b, but for WTC11.

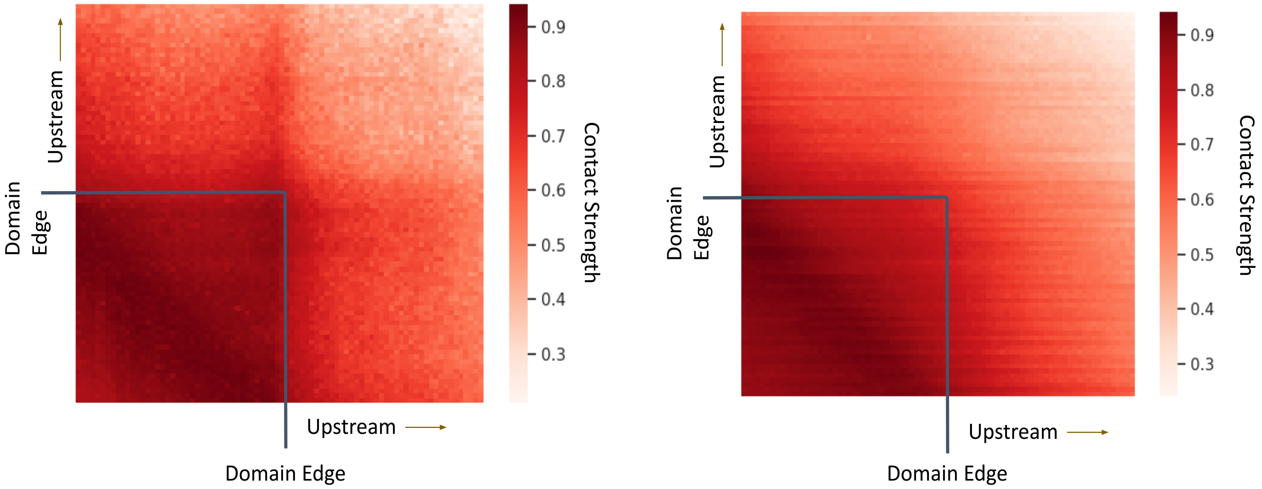

Figure 2: **Hi-C-LSTM predictions near CTCF interaction dots.** The edges of most domains are usually dotted by the CTCF transcription factor binding and holding the domains together. This results in increased contacts at the very edges of domains compared to their vicinity. **a)** We show the average contact strength at the edges of medium sized domains (300Kbp) and their vicinity in the observed Hi-C. We go up to 50Kbp within the domain and show 50Kbp upstream on both sides. The CTCF interaction dots are visible at the domain edges. **b)** However, the same is not observed for predicted contacts from Hi-C-LSTM. We see that the domain edges are a bit smudged because of the sequential streaks in the Hi-C-LSTM predictions, but the model is able to capture the distinction between the contacts within and outside the domain. Although we lose CTCF interaction dots in the reconstructed output, we are able to accurately predict domain boundaries and CTCF binding sites using Hi-C-LSTM representations.

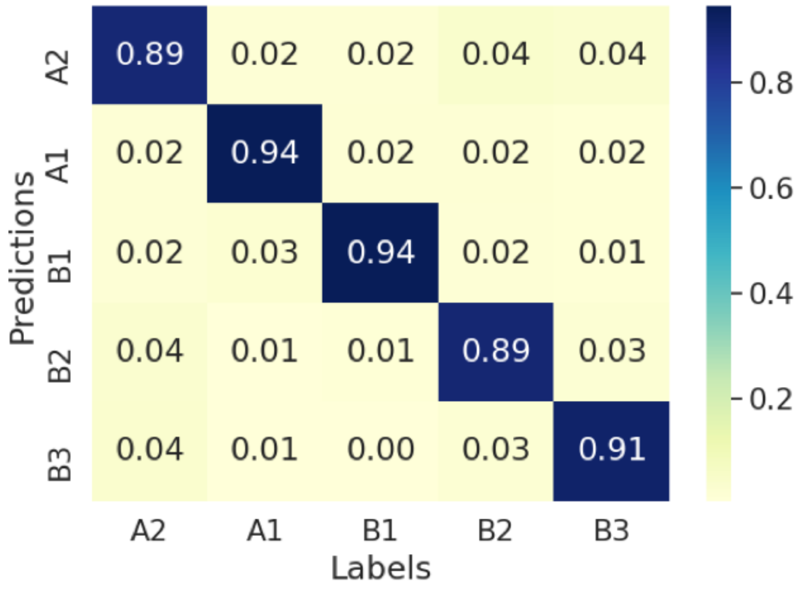

Figure 3: **Subcompartment Confusion Matrix.** The Hi-C-LSTM representations are used to classify subcompartments in the Genome and the resulting confusion matrix is plotted. The x-axis shows the subcompartment labels. The y-axis shows the subcompartment predictions by the XGBoost classifier, and the values in the confusion matrix are true prediction percentages. All subcompartments achieve a true prediction percentage $>\approx 90\%$. The A1 and B1 subcompartments achieve the highest true predictions while the other subcompartments follow closely.

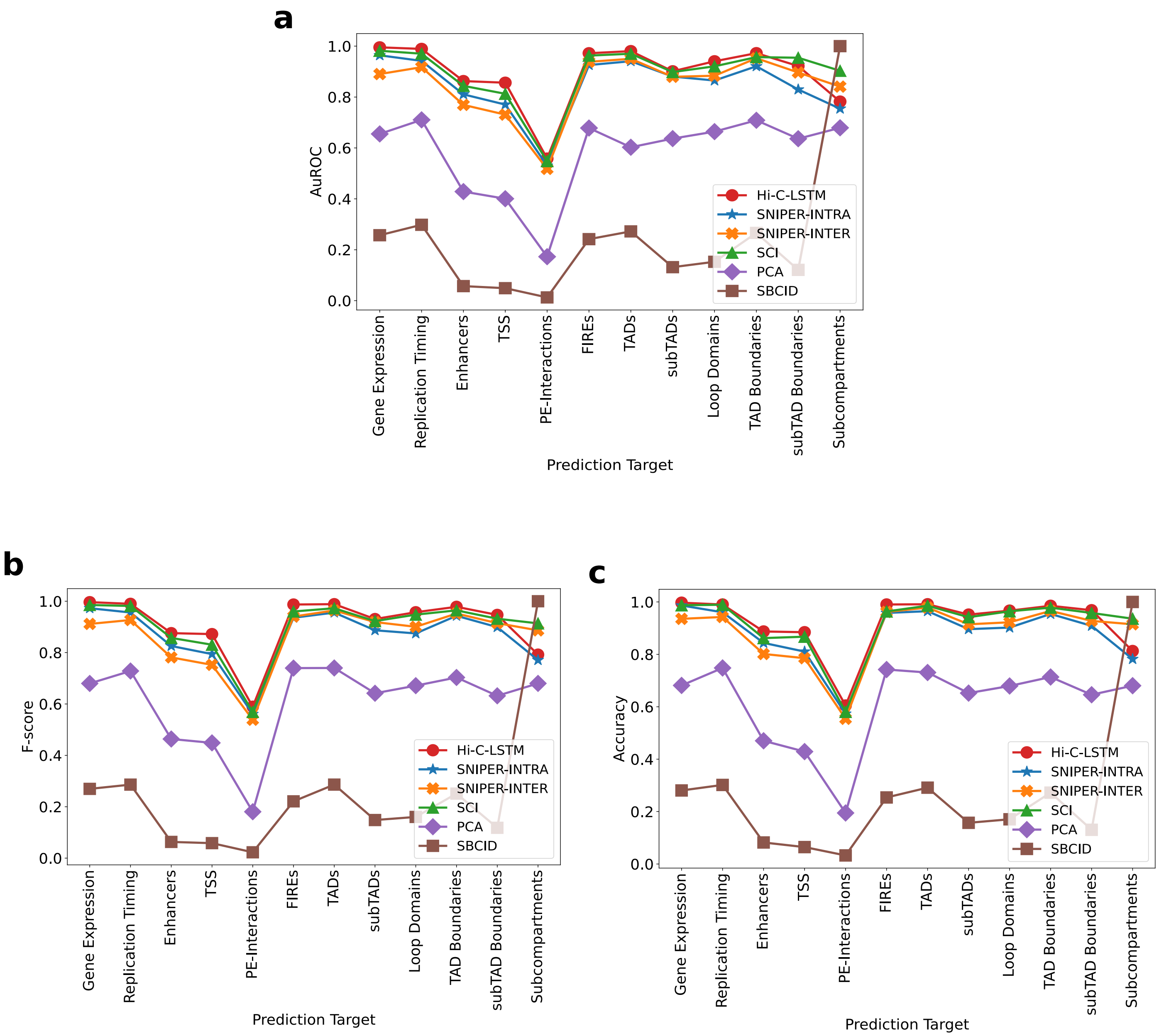

Figure 4: **Additional classification metrics in GM12878.** Metrics for gene expression, replication timing, enhancers, transcription start sites (TSSs), promoter-enhancer interactions(PEIs), frequently interacting regions (FIREs), topologically associating domains (TADs), subTADs, loop domains, TAD boundaries, subTAD boundaries, and subcompartments. **a, b, c)** The area under the receiver operating characteristic curve (AuROC), F-score, and Accuracy for different prediction targets. The y-axis shows the metrics, the x-ticks refer to the prediction targets, and the legend shows the different methods compared with.

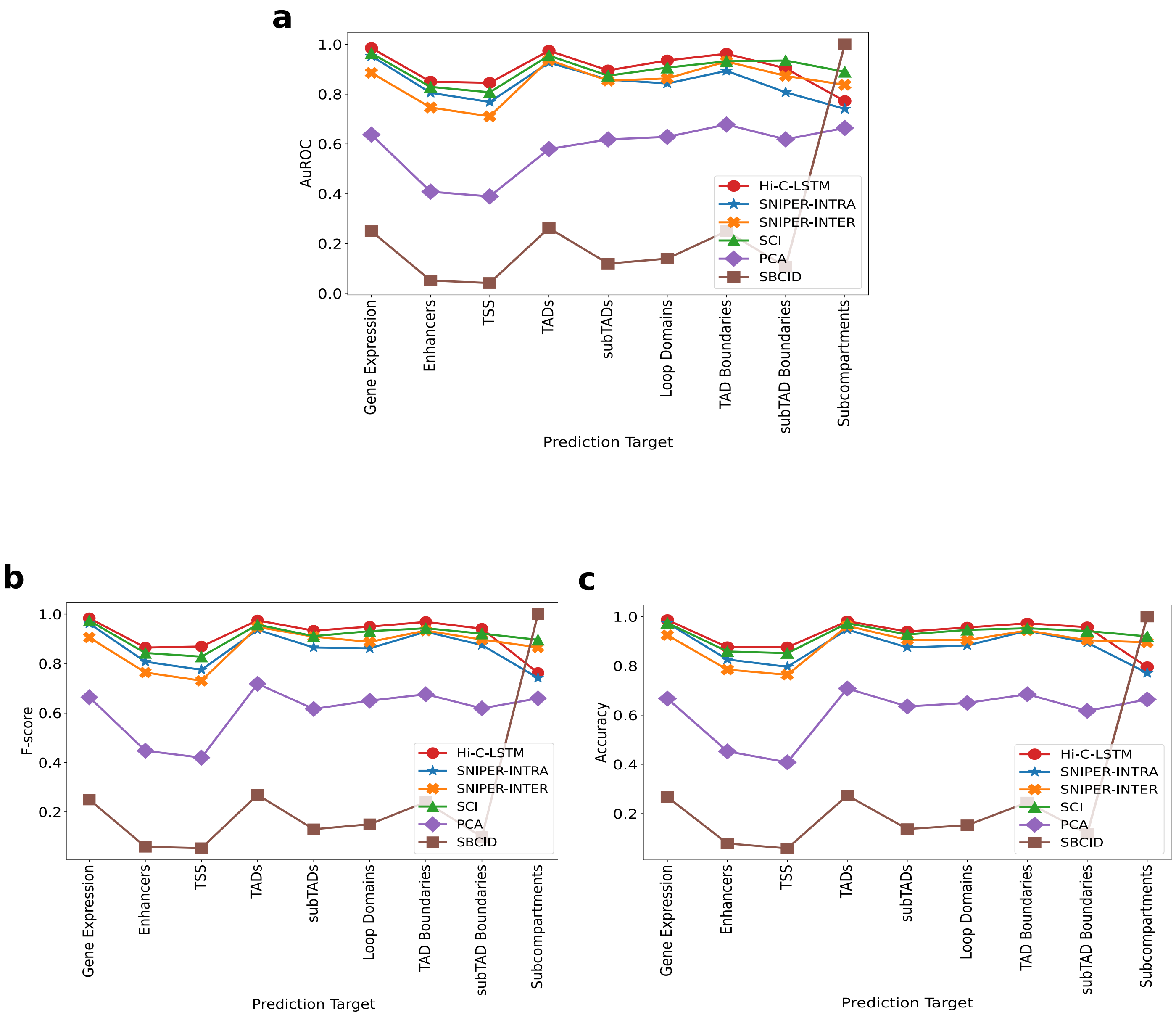

Figure 5: **Additional classification metrics in H1-hESC.** Metrics for gene expression, enhancers, transcription start sites (TSSs), topologically associating domains (TADs), subTADs, loop domains, TAD boundaries, subTAD boundaries, and subcompartments. **a, b, c)** The area under the receiver operating characteristic curve (AuROC), F-score, and Accuracy for different prediction targets. The y-axis shows the metrics, the x-ticks refer to the prediction targets, and the legend shows the different methods compared with.

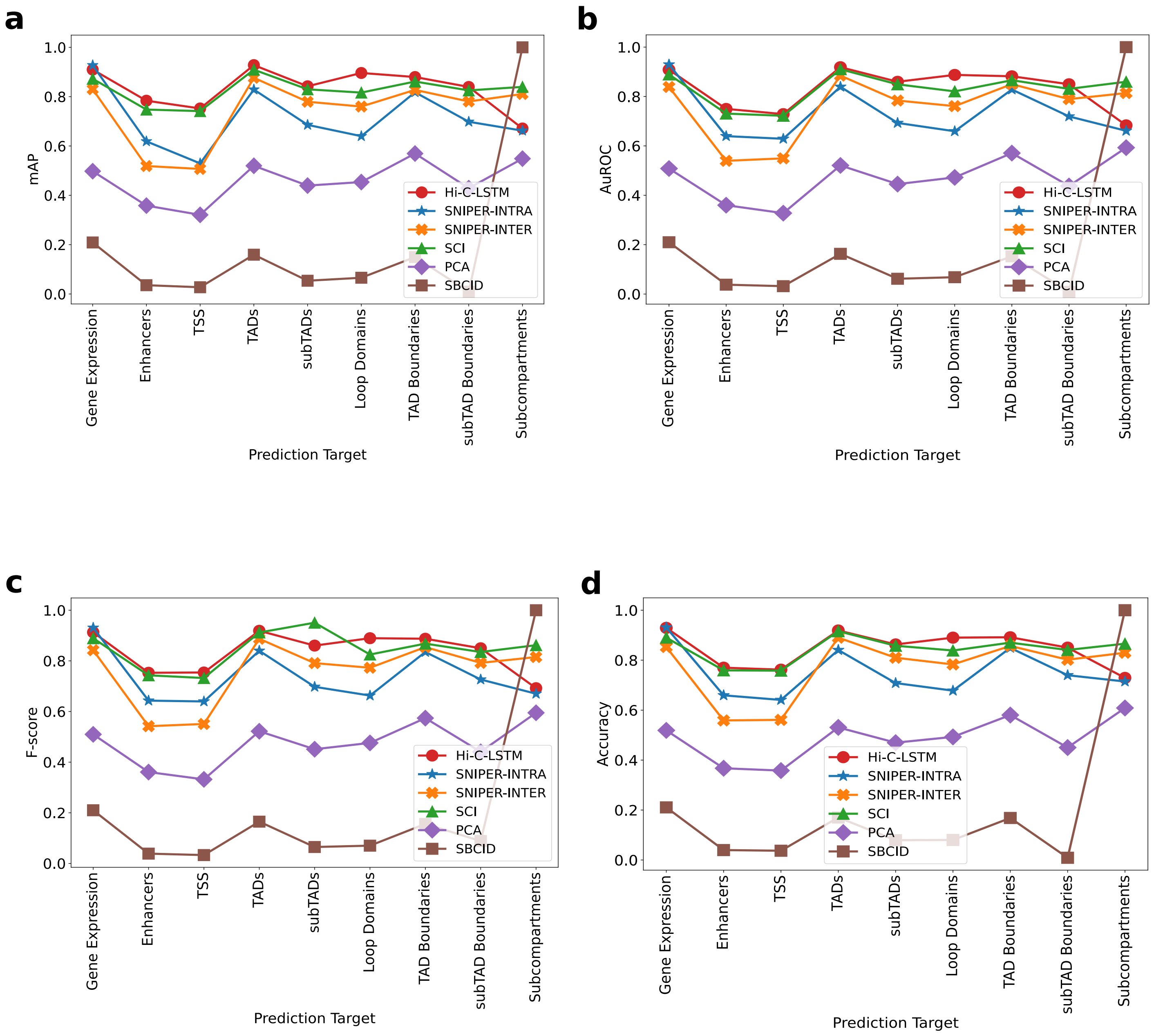

Figure 6: **Additional classification metrics in HFF-hTERT.** Metrics for gene expression, enhancers, transcription start sites (TSSs), topologically associating domains (TADs), subTADs, loop domains, TAD boundaries, subTAD boundaries, and subcompartments. **a, b, c, d)** mean average precision (mAP), The area under the receiver operating characteristic curve (AuROC), F-score, and Accuracy for different prediction targets. The y-axis shows the metrics, the x-ticks refer to the prediction targets, and the legend shows the different methods compared with.

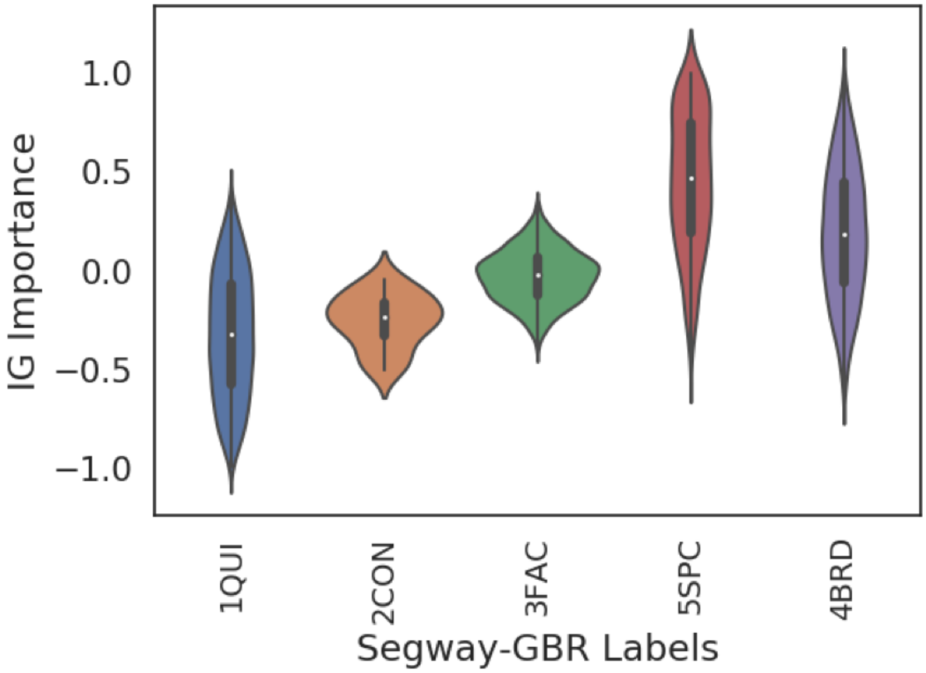

Figure 7: **Segway-GBR Feature Importance.** Segway, by itself, cannot handle chromatin conformation data, however, when coupled with graph-based regularization (GBR), a method that encourages positions that are close in 3D space to occupy the same type of domain by including pairwise prior information during genome annotation, a model revealing known chromatin domains is obtained. Segway GBR gives five types of domains: quiescent (QUI), constitutive heterochromatin (CON), facultative heterochromatin (FAC), broad expression (BRD), and specific expression (SPC). Quiescent, constitutive and facultative domains are repressive. Broad and specific expression domains are active. Unlike BRD domains, genes present in a SPC domain are highly expressed compared to their mean in all cell types, hinting that SPC domains might be highly activating. The plot of aggregated feature importance scores shows largely positive values for SPC and BRD domains and largely negative values for QUI and CON domains, while FAC domains exhibit a bit of both. Both the highly activating nature of SPC domains and the repressive nature of QUI and CON domains is thereby validated. Violin plot presents summary statistics where the white dot is the median, thick gray bar is the inter-quartile range, and thin gray line is the rest of the distribution. Kernel density estimation is shown on either side of the line. Sample size for the domains are calculated genome wide by considering all observations of domains according to domain specific data.

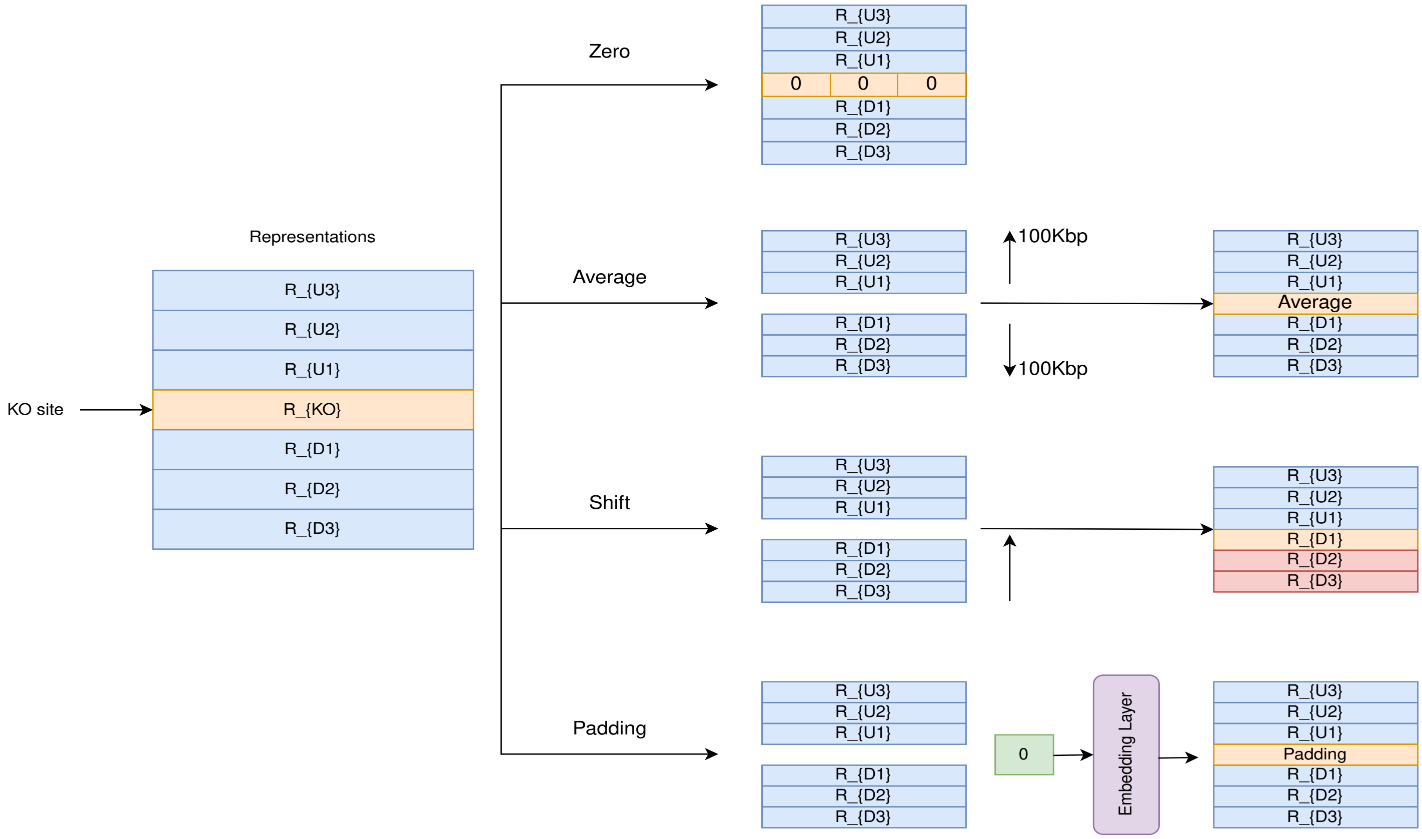

Figure 8: **Different ways to perform knockout.** (1) Zero representations: replace the representation at the knockout site with the zero representations. 2) Average representation: replace the representation at the knockout site with the average of the representations 100Kbp upstream and downstream of the knockout site. 3) Shift representations: remove the representation at the knockout site and shift all downstream representations upward. 4) Padding representation: replace the representation at the knockout site with the representation obtained from the embedding layer for the input indice zero (zero is considered as padding).

P-value tables for feature attribution

Table 1: **P-values from the two-sided T-tests of feature attribution score samples for various elements.** Feature scores from each element were tested with scores obtained by sampling randomly from the other elements. **a)** P-values for 3 categories of inactive elements from segway, namely, Repressed, Dead, and Low Regions. **b)** P-values for 6 categories of active elements, namely, Gene Body, TF, Enhancers, TSS, FAIRE, and FIREs. **c)** P-values for pairs of elements. Feature scores from one group were tested with scores from the other group. **d)** P-values for 4 transcriptions factors (TFs), namely, ZNF143, FOXG1, SOX2, and XBP1. Feature scores from each TF were tested with scores obtained by sampling randomly from the other TFs.

(a)

Inactive Elements	P-value
Repressed	3.45e-9
Dead	2.79e-7
Low	1.93e-8

(b)

Active Elements	P-value
Gene Body	7.04e-5
TF	6.28e-6
Enhancer	2.18e-4
TSS	8.61e-4
FAIRE	6.27e-4
FIREs	8.17e-5

(c)

Element 1	Element 2	P-value
CTCF (weak)	CTCF (strong)	0.08
TAD Boundaries (CTCF+)	TAD Boundaries (CTCF-)	4.62e-4
Loop Domains	Non-loop Domains	8.27e-5
CTCF+Cohesin (loop)	CTCF+Cohesin (Non-loop)	1.26e-5

(d)

Transcription Factors	P-value
ZNF143	0.0026
FOXG1	0.0071
SOX2	6.48e-4
XBP1	0.0063

Datasets

Dataset	Link
GM12878 Hi-C	https://www.ncbi.nlm.nih.gov/geo/query/acc.cgi?acc=GSE63525
H1-hESC Hi-C	https://data.4dnucleome.org/experiment-set-replicates/4DNES2M5JIGV
WTC11 Hi-C	https://data.4dnucleome.org/experiment-set-replicates/4DNESPDEZNWX
HFF-hTERT Hi-C	https://data.4dnucleome.org/experiment-set-replicates/4DNESVUMGLG2
GM12878 Hi-C (300M)	https://data.4dnucleome.org/experiment-set-replicates/4DNESJFTAURO
GM12878 Hi-C (216M)	https://data.4dnucleome.org/experiment-set-replicates/4DNESLQG7ZKJ
Juicer Tools	https://github.com/aidenlab/juicer/wiki/Juicer-Tools-Quick-Start
RNA-seq (GM12878, H1-hESC, HFF-hTERT)	https://egg2.wustl.edu/roadmap/data/byDataType/rna/expression
Promoter-Enhancer Interactions (GM12878)	https://github.com/shwhalen/targetfinder
Replication Timing (GM12878)	https://www2.replicationdomain.com
Enhancers (GM12878, H1-hESC, HFF-hTERT)	https://fantom.gsc.riken.jp/5
Transcription Start Sites (GM12878, H1-hESC, HFF-hTERT)	https://www.encodeproject.org/files/ENCFF140PCA
Loop Domains and Subcompartments (GM12878)	https://www.ncbi.nlm.nih.gov/geo/query/acc.cgi?acc=GSE63525
Segway Labels	https://segway.hoffmanlab.org
CTCF and Cohesin Peaks (GM12878)	https://www.encodeproject.org
CTCF Motif	https://meme-suite.org/meme/doc/fimo.html
Transcription Factor Binding Sites	http://humantfs.ccbr.utoronto.ca
Pseudo-Bulk Single-Cell Hi-C	https://www.ncbi.nlm.nih.gov/geo/query/acc.cgi?acc=GSM2254215

Table 2

Running Time

Method	Training time in hrs	Test time in mns
Hi-C-LSTM	2.549	14.37
SNIPER	1.805	8.61
SCI	2.438	12.14

Table 3: **Running times of different methods for the whole genome.** The training time is given in hours and the test time is given in minutes. As SNIPER uses only a feedforward neural network without considering a sequential input, it has much better training and test running times compared to SCI and Hi-C-LSTM. Hi-C-LSTM running time is comparable to SCI both during training and inference. SCI uses a graph embedding algorithm called LINE (with parameters mentioned in SCI) that roughly takes the same time to run as an LSTM with frame length on the order of 100. We verify this by increasing LSTM frame length and observe that both training and inference times get worse with increasing frame length.

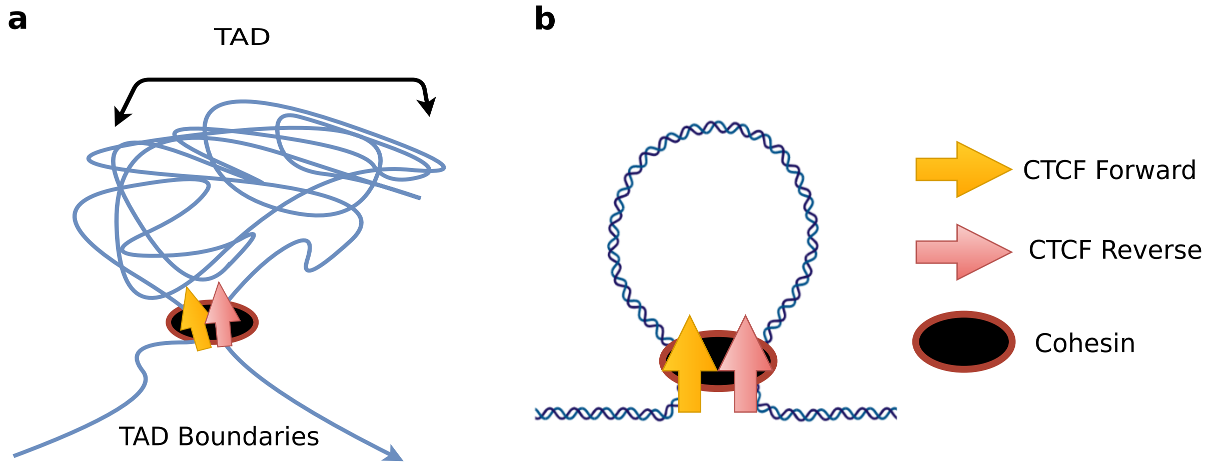

Figure 9: **The arrangement of CTCF and Cohesin at the edges of domains. a)** Both the forward and reverse CTCF motifs align at the edges of topologically associating domains (TADs), held together by the ring formed by Cohesin subunits. Densely interacting genomic regions give rise to TADs and neighbouring TADs are insulated from each other by the combined action of CTCF and Cohesin at TAD boundaries. **b)** While TADs are larger structural elements, smaller loop domains are also important structural units formed by chromatin folding. The edges of loop domains are also held together by the aforementioned tandem action of CTCF binding and Cohesin ring enclosing the edges of loop domains.

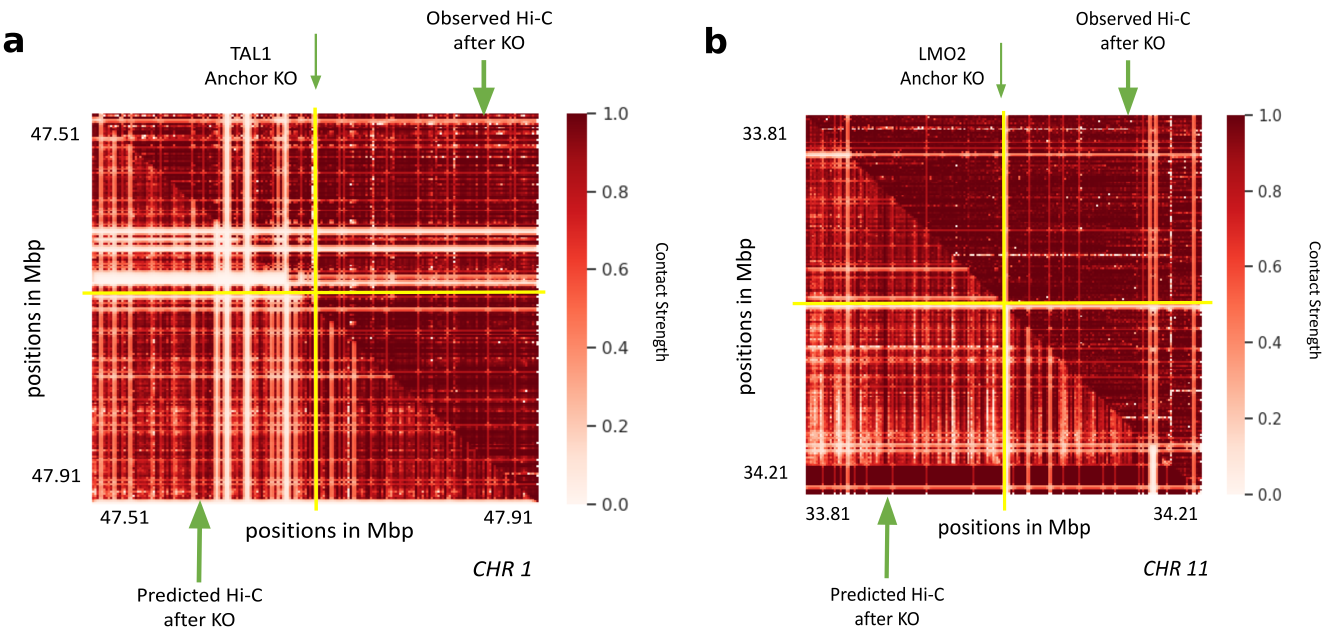

Figure 10: **Hi-C-LSTM simulated post-deletion Hi-C compared with observed post-deletion Hi-C. a)** Observed Hi-C contacts after TAL1 deletion (upper-triangle), and predicted Hi-C contacts after TAL1 deletion (lower-triangle). **b)** Observed Hi-C contacts after LMO2 deletion (upper-triangle), and predicted Hi-C contacts after LMO2 deletion (lower-triangle).

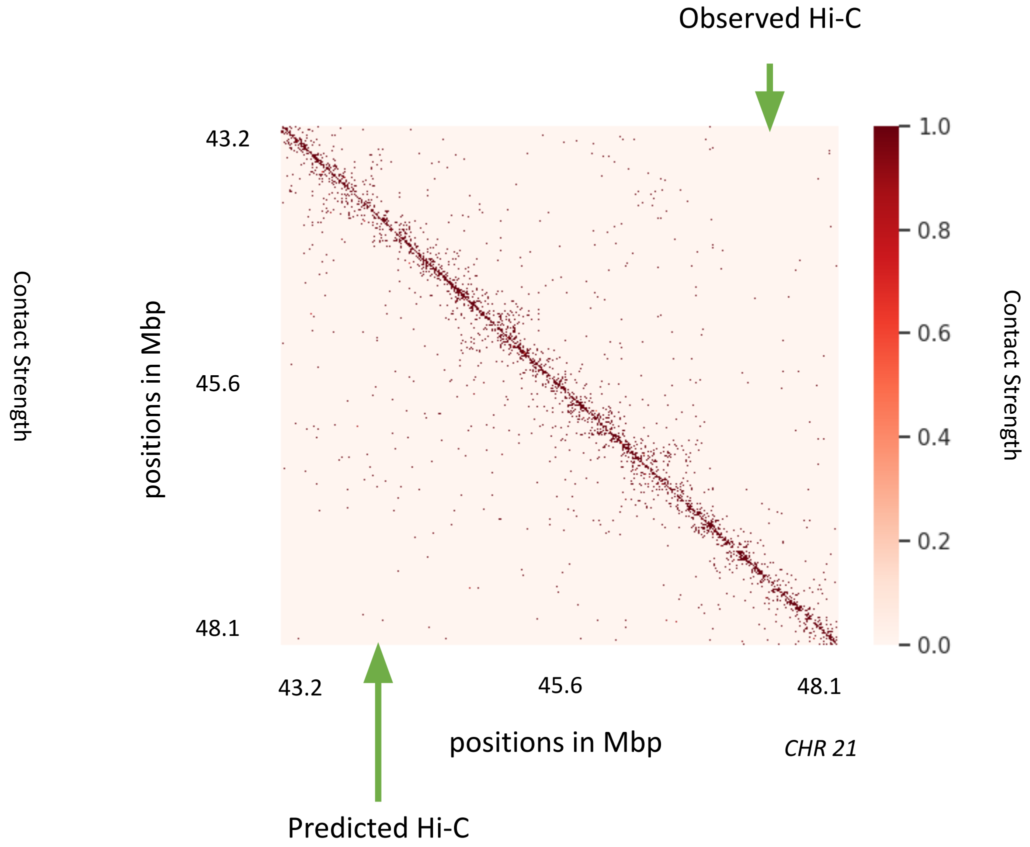

Figure 11: **Hi-C-LSTM applied to pseudo-bulk single-cell Hi-C (scHi-C) data.** A selected portion of the observed scHi-C map (upper-triangle) [1] and the predicted scHi-C map (lower-triangle). The portion is selected from chromosome 21, between 43.2 Mbp to 48.1 Mbp. Hi-C-LSTM does a good job of recapitulating the sparse structures in the observed scHi-C. The ability of Hi-C-LSTM to handle such sparse data alludes to its prowess in reconstructing data from minimal number of data points.

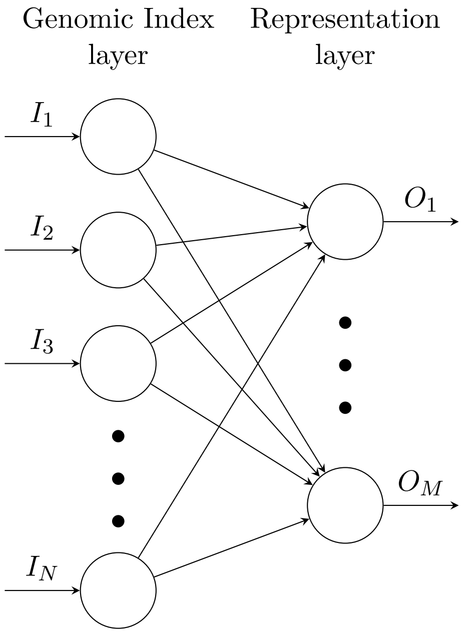

Figure 12: **An illustration of the embedding neural network layer.** N is the chromosome length, and M is the representation size. The PyTorch Embedding layer we use is essentially just a Linear layer. We could alternatively define it as a linear layer where the number of inputs corresponds to the chromosome length and the number of outputs corresponds to the representation size. Here, each genomic index is represented as a one-hot vector, where the length of the vector is equal to the chromosome length, with a 1 in a unique position, compared to all other genomic indices. The PyTorch Embedding layer simplifies this by requiring just the position index instead of the big one-hot vector.

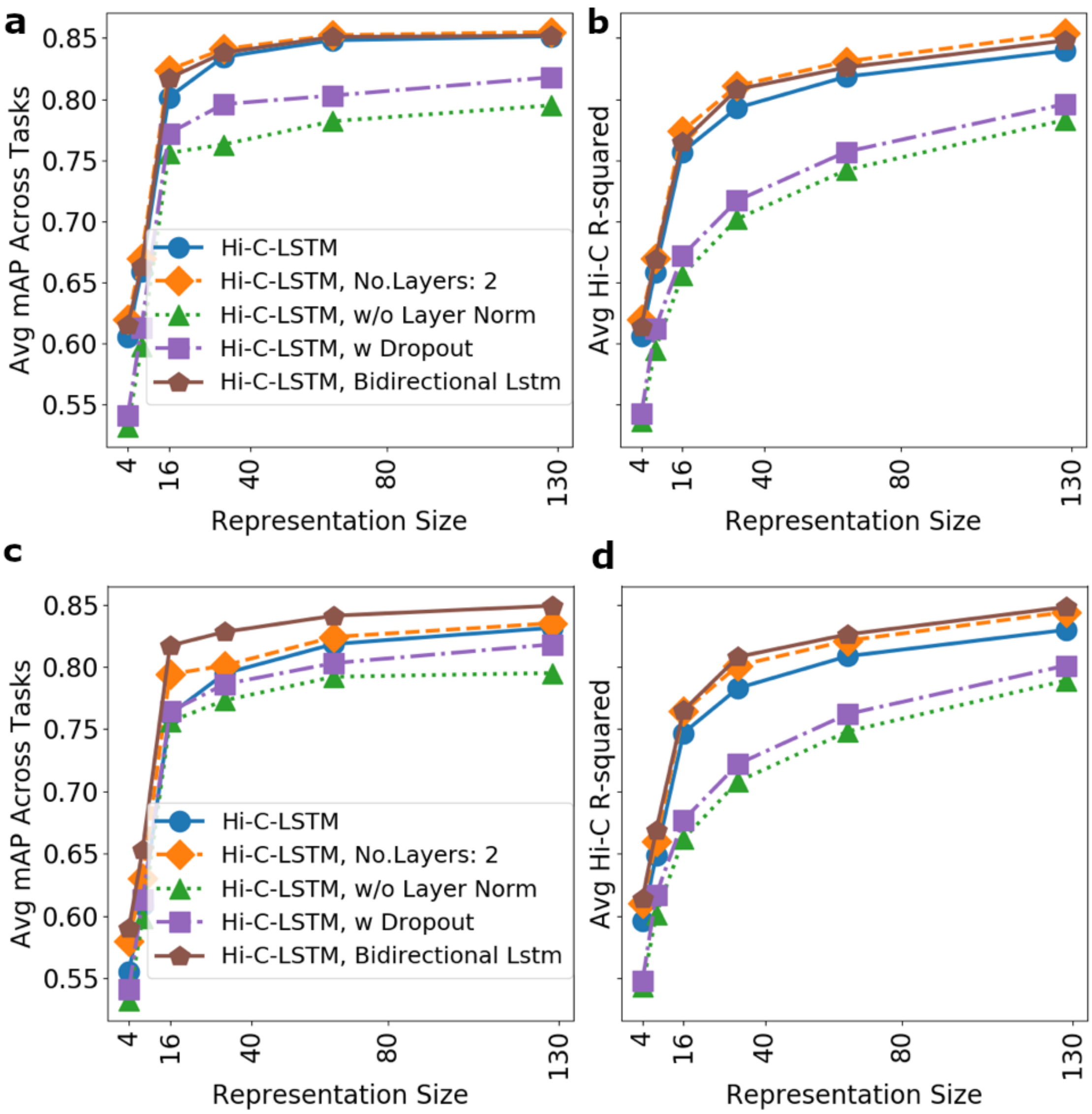

Figure 13: **Ablation Results.** To choose the representation size of our model, we performed an ablation analysis. **a)** Average mAP across downstream tasks (y-axis) with the Hi-C-LSTM model (single layer, unidirectional LSTM with layer norm in the absence of dropout) and its ablations with increasing representation size (x-axis) for odd chromosomes. **b)** The average Hi-C R-squared (y-axis) with representation size (x-axis) for odd chromosomes. The ablations of the model are shown in the legend with different markers and colors. The performance trend is preserved across a and b, which is indicative of the fact that faithfully creating the Hi-C matrix helps in performing well across classification tasks. **c,d)** Similar trend is seen for even chromosomes. In both a,b and c,d, the Hi-C R-squared increases considerably with hidden size, however, we notice an elbow at an embedding size of 16 for average classification mAP and thus set our embedding size to that value as a trade-off. It is interesting to see that a low-dimensional Hi-C-LSTM representation is able to successfully create the H-C matrix as well as do well across classification tasks.

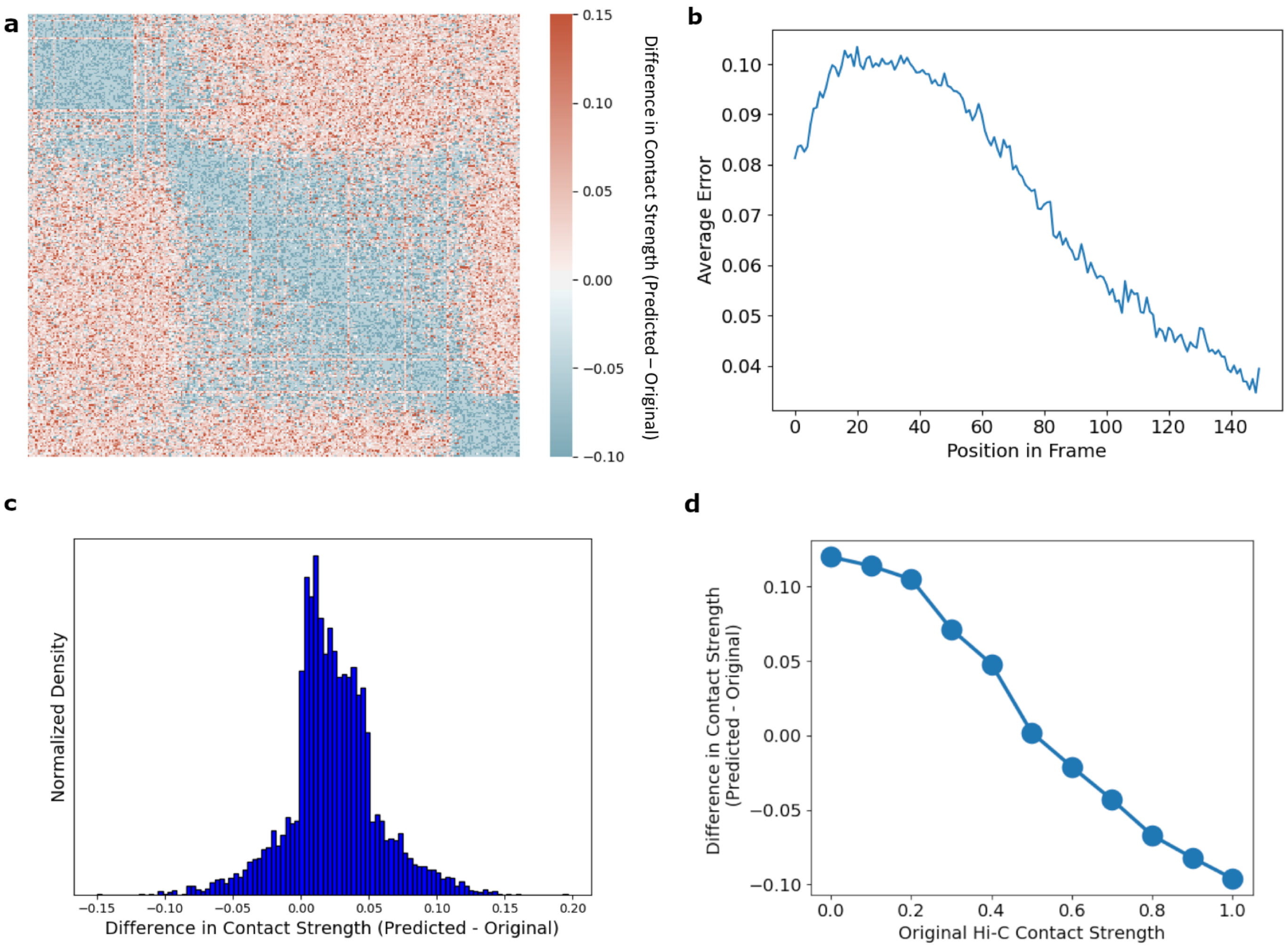

Figure 14: **Salient characteristics of Hi-C-LSTM predictions.** Analysed by comparing predictions with original Hi-C values and visualizing their range. **a)** The difference in contact strength between the predicted Hi-C map and original Hi-C map for a selected genomic region between 41.4 to 41.6 Mbp. **b)** Average prediction error within each frame. We see that error is high at the beginning of the frame and improves towards the end of the frame, explaining the discontinuity in our predictions at the start of each frame. **c)** The histogram of the difference between predicted and original Hi-C values. The y-axis shows the normalized density and the x-axis refers to the difference in contact strength between predicted and original Hi-C. The plot reveals the low spread and slight positive skew of prediction errors. **d)** The variation of difference in predicted and original Hi-C (y-axis) across the range of original Hi-C values (x-axis). Its seen that the errors go from positive to negative as values of original Hi-C increase, which can also be seen in a, pointing to the Hi-C-LSTM over-predicting low values and under-predicting high values of Hi-C.

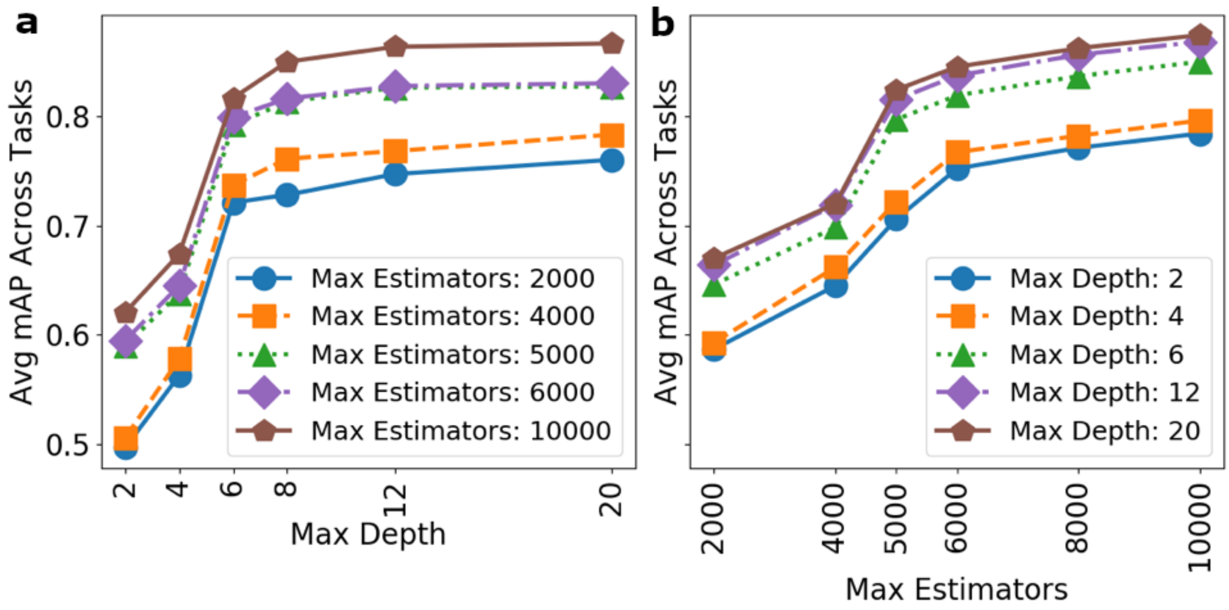

Figure 15: **XGBoost Parameters.** To choose the maximum depth and the maximum number of estimators of the XGBoost classification framework, we performed an ablation analysis with odd chromosomes as the training set and even chromosomes as the test set. The test results are shown here. **a)** Average mAP across downstream tasks (y-axis) using the Hi-C-LSTM representations with increasing maximum depth (x-axis). The legend shows the maximum number of estimators used in each case. As the elbow in a is seen at a maximum depth of 6 for all values of maximum estimators, we set our maximum depth to this value, achieving optimum gains in performance and run time. **b)** The average mAP across downstream tasks (y-axis) with increasing maximum number of estimators (x-axis). The legend shows the maximum depth used in each case. In b, the elbow is clearly seen at 5000 maximum estimators for different maximum depths. Following this, we set our maximum number of estimators to 5000.

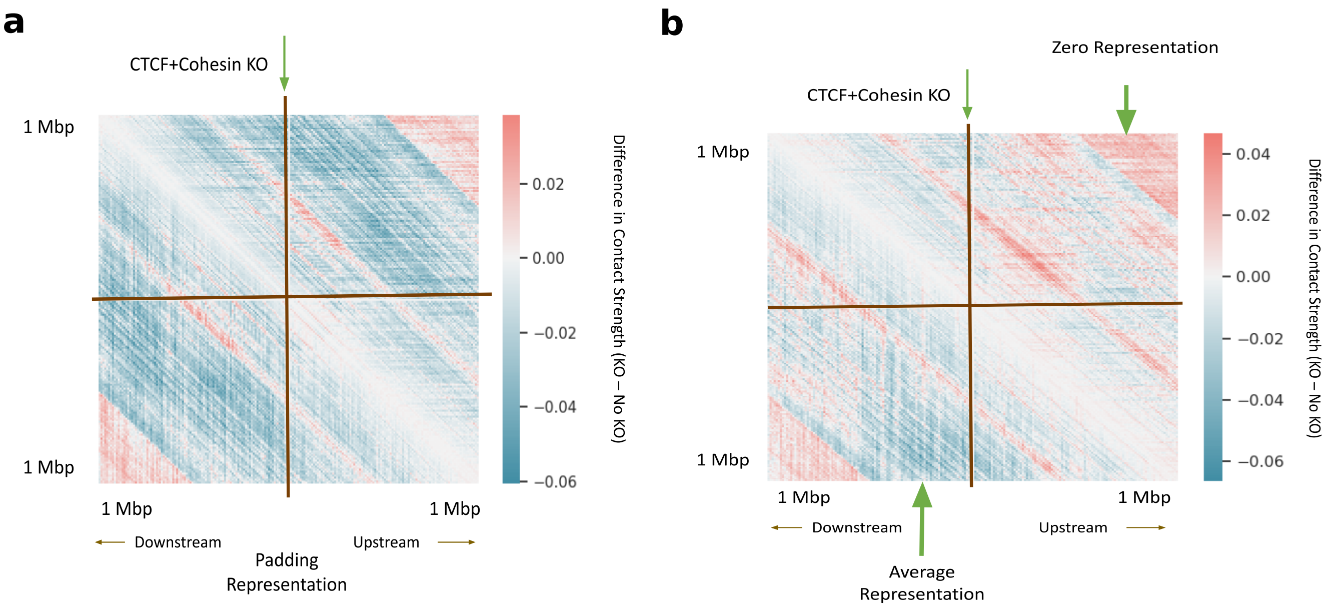

Figure 16: **The average difference in predicted Hi-C contact strength using padding, zero, and average representations.** Difference calculated between CTCF+Cohesin knockout and no knockout in a 2Mb window. **a)** Padding representation: replace the representation at the knockout site with the representation obtained from the embedding layer for the input indice zero (zero is considered as padding). **b)** Zero representation (upper-triangle): replace the representation at the knockout site with the zero representations, and average representation (lower-triangle): replace the representation at the knockout site with the average of the representations 100Kbp upstream and downstream of the knockout site.

References

[1] Ramani, V. et al. Massively multiplex single-cell Hi-C. *Nature methods* **14,** 263-266 (2017).
