# Supplementary material for: Learning representations of chromatin contacts using a recurrent neural network identifies genomic drivers of conformation
Source: Nat Commun. 2022 Jun 28;13:3704. doi: 10.1038/s41467-022-31337-w (PMC9240038; doi:10.1038/s41467-022-31337-w)
Supplement: Supplementary file 2 — Reporting Summary [file 41467_2022_31337_MOESM2_ESM.pdf]

nature portfolio

Corresponding author(s): Maxwell W. Libbrecht, Kevin B. Dsouza

Last updated by author(s): Jun 8, 2022

Reporting Summary

Nature Portfolio wishes to improve the reproducibility of the work that we publish. This form provides structure for consistency and transparency in reporting. For further information on Nature Portfolio policies, see our Editorial Policies and the Editorial Policy Checklist.

Statistics

For all statistical analyses, confirm that the following items are present in the figure legend, table legend, main text, or Methods section.

n/a	Confirmed	
☐	☒	The exact sample size (n) for each experimental group/condition, given as a discrete number and unit of measurement
☒	☐	A statement on whether measurements were taken from distinct samples or whether the same sample was measured repeatedly
☐	☒	The statistical test(s) used AND whether they are one- or two-sided<br>*Only common tests should be described solely by name; describe more complex techniques in the Methods section.*
☒	☐	A description of all covariates tested
☒	☐	A description of any assumptions or corrections, such as tests of normality and adjustment for multiple comparisons
☐	☒	A full description of the statistical parameters including central tendency (e.g. means) or other basic estimates (e.g. regression coefficient) AND variation (e.g. standard deviation) or associated estimates of uncertainty (e.g. confidence intervals)
☐	☒	For null hypothesis testing, the test statistic (e.g. F, t, r) with confidence intervals, effect sizes, degrees of freedom and P value noted<br>*Give P values as exact values whenever suitable.*
☒	☐	For Bayesian analysis, information on the choice of priors and Markov chain Monte Carlo settings
☐	☒	For hierarchical and complex designs, identification of the appropriate level for tests and full reporting of outcomes
☒	☐	Estimates of effect sizes (e.g. Cohen's d, Pearson's r), indicating how they were calculated

Our web collection on statistics for biologists contains articles on many of the points above.

Software and code

Policy information about availability of computer code

Data collection

1. Juicer Tools V1.6 (https://github.com/aidenlab/juicer/wiki/Juicer-Tools-Quick-Start) was used to extract text files from downloaded Hi-C files
2. For H1-hESC and HFF-hTERT, Loop Domains were obtained by running HICCUPS (Rao et al. 2014) and Subcompartments were obtained by running Gaussian HMM (Rao et al. 2014)
3. MEME suite V 5.3.3 and FIMO were used to get CTCF motif instances
4. For GM12878, subTADs were obtained by running GMAP (Yu et al. 2017). For H1-hESC and HFF-hTERT, both TADs and subTADs were obtained by running GMAP (Yu et al. 2017)
5. The validPairs file (GEO accession GSM2254215) was used to filter single cell Hi-C data based on chromosome, positions and barcodes. The number of reads aligned to hg19 corresponding to each distinct pair of bar codes was obtained using the percentages file (GEO accession GSM2254215).
6. Sniper representations were obtained by running Sniper (https://github.com/ma-compbio/SNIPER). SCI representations were obtained by running SCI (https://github.com/TheJackso\\nLaboratory/sci)
7. All other data used in the study was obtained directly from hosted servers (Refer to the Data section in this document or the Data Availability Statement in the manuscript)

Rao et al. 2014: Rao, S. S. et al. A 3D map of the human genome at kilobase resolution reveals principles of chromatin looping. Cell 159, 1665-80 (2014).
Yu et al. 2017: Yu, W., He, B. Tan, K. Identifying topologically associating domains and subdomains by Gaussian mixture model and proportion test. Nature communications 8, 1-9 (2017).
GEO accession GSM2254215: https://www.ncbi.nlm.nih.gov/geo/query/acc.cgi?acc=GSM2254215

Data analysis | The complete code used to analyze data in the study can be found at: https://github.com/kevinbdsouza/Hi-C-LSTM

For manuscripts utilizing custom algorithms or software that are central to the research but not yet described in published literature, software must be made available to editors and reviewers. We strongly encourage code deposition in a community repository (e.g. GitHub). See the Nature Portfolio guidelines for submitting code & software for further information.

Data

Policy information about availability of data

All manuscripts must include a data availability statement. This statement should provide the following information, where applicable:

- Accession codes, unique identifiers, or web links for publicly available datasets
- A description of any restrictions on data availability
- For clinical datasets or third party data, please ensure that the statement adheres to our policy

1. The Hi-C data for GM12878 was acquired using the GEO accession number GSE63525 (https://www.ncbi.nlm.nih.gov/geo/query/acc.cgi?acc=GSE63525). The Hi-C data for other tier 1 cell types was acquired from the 4DN Data Portal, like H1-hESC (https://data.4dnucleome.org/experiment-set-replicates/4DNES2M5JIGV/), WTC11 (https://data.4dnucleome.org/experiment-set-replicates/4DNESPDEZNWX/), and HFF-hTERT (https://data.4dnucleome.org/experiment-set-replicates/4DNESVUMGLG2/). The Hi-C data for GM12878 with lower read depths were also downloaded from the 4DN Data Portal, like 300M (https://data.4dnucleome.org/experiment-set-replicates/4DNESJFTAURO/) and 216M (https://data.4dnucleome.org/experiment-set-replicates/4DNESLQG7ZKJ/).

2. The intra-chromosomal Hi-C data set text file on the hg19 human reference genome assembly was obtained at 10Kb resolution with KR (Balanced) normalization using juicer tools (https://github.com/aidenlab/juicer/wiki/Juicer-Tools-Quick-Start)

3. RNA-seq data for GM12878, H1-hESC, and HFF-hTERT was obtained from the Roadmap Consortium (https://egg2.wustl.edu/roadmap/data/byDataType/rna/expression/). For GM12878, H1-hESC, and HFF-hTERT, locations of known enhancers and transcription start sites (TSSs) were obtained from FANTOM (https://fantom.gsc.riken.jp/5) and ENCODE (https://www.encodeproject.org/files/ENCFF140PCA) respectively.

4. For GM12878, we defined promoter-enhancer interactions (PEI) as the ones that were used to train TargetFinder (https://github.com/shwhalen/targetfinder). For GM12878, Frequently interacting region (FIRE) scores at 40Kbp resolution were downloaded from the additional material of Schmitt et. al 2016. For GM12878, the replication timing data was downloaded from Replication Domain (https://www2.replicationdomain.com) at 40Kbp resolution.

5. For GM12878, Loop Domains and Subcompartments were obtained using GEO accession GSE63525 (https://www.ncbi.nlm.nih.gov/geo/query/acc.cgi?acc=GSE63525). For H1-hESC and HFF-hTERT, Loop Domains were obtained by running HICCUPS (Rao et al. 2014) and Subcompartments were obtained by running Gaussian HMM (Rao et al. 2014).

6. Segway and Segway-GBR labels were obtained from Hoffmanlab (https://segway.hoffmanlab.org) and Noblelab (https://noble.gs.washington.edu/proj/gbr) respectively

7. CTCF, Cohesin peak calls for GM12878 were downloaded from ENCODE} (https://www.encodeproject.org). The CTCF orientations were obtained by using the CTCF motif from the MEME suite version 5.3.3 (https://meme-suite.org/meme/doc/fimo.html) and running FIMO (Grant et al. 2011) to get the motif instances. Other Transcription Factor binding sites (TFBS) for the feature importance evaluation were downloaded from the The Human Transcription Factors repository (http://humantfs.ccbr.utoronto.ca/).

8. For GM12878, Topologically-associating domains (TADs) were downloaded from TADKB (Liu et al. 2019) and subTADs were obtained by running GMAP (Yu et al. 2017). For H1-hESC and HFF-hTERT, both TADs and subTADs were obtained by running GMAP (Yu et al. 2017).

9. For our duplication experiment, we obtained the duplicated Hi-C for the 2.1 Mbp region between 67.95 Mbp to 70.08 Mbp in chromosome 7 from Melo et al. 2020. For our anchor deletion experiment, we obtained the 5C data for the TAL1 and LMO2 fragments in chromosome 1 and 11 from Hnisz et al. 2016.

10. Pseudo-bulk single-cell Hi-C (scHi-C) data was downloaded using GEO accession number GSM2254215 (https://www.ncbi.nlm.nih.gov/geo/query/acc.cgi?acc=GSM2254215)

Schmitt et. al 2016: Schmitt, A. D. et al. A compendium of chromatin contact maps reveals spatially active regions in the human genome. Cell reports 17, 2042-59 (2016).
Rao et. al 2014: Rao, S. S. et al. A 3D map of the human genome at kilobase resolution reveals principles of chromatin looping. Cell 159, 1665-80 (2014).
Grant et. al: Grant, C. E., Bailey, T. L. Noble, W. S. FIMO: scanning for occurrences of a given motif. Bioinformatics 27, 1017-1018 (2011).
Liu et. al 2019: Liu, T. et al. TADKB: Family classification and a knowledge base of topologically associating domains. BMC genomics 20, 1-17 (2019).
Yu et. al 2017: Yu, W., He, B. Tan, K. Identifying topologically associating domains and subdomains by Gaussian mixture model and proportion test. Nature communications 8, 1-9 (2017).
Melo et al. 2020: Melo, U. S. et al. Hi-C identifies complex genomic rearrangements and TAD-shuffling in developmental diseases. The American Journal of Human Genetics 106, 872-884 (2020).
Hnisz et al. 2016: Hnisz, D. et al. Activation of proto-oncogenes by disruption of chromosome neighborhoods. Science 351, 1454-1458 (2016).

Field-specific reporting

Please select the one below that is the best fit for your research. If you are not sure, read the appropriate sections before making your selection.

☒ Life sciences ☐ Behavioural & social sciences ☐ Ecological, evolutionary & environmental sciences

For a reference copy of the document with all sections, see nature.com/documents/nr-reporting-summary-flat.pdf

Life sciences study design

All studies must disclose on these points even when the disclosure is negative.

Sample size	Sample size for the genomic elements used in the study are calculated genome wide by considering all observations of elements according to element specific data without excluding any particular region.
Data exclusions	No data was excluded in the study.
Replication	The analyses and results in the paper were made to adhere to the Silver Reproducibility Standard as defined by Heil et al (Heil, B. J. et al. Reproducibility standards for machine learning in the life sciences. Nature Methods, 18, 1132-1135, 2021); in particular, a user can deterministically acquire all dependencies and run all analyses. Some of the measures taken to verify the reproducibility were running the same experiment multiple times, making the software modular and configurable, and creating a separate pipeline for each downstream analysis.
Randomization	Randomization not relevant to the study because of the computational nature of the study, with no experimental groups.
Blinding	Blinding not relevant to the study because of the computational nature of the study.

Reporting for specific materials, systems and methods

We require information from authors about some types of materials, experimental systems and methods used in many studies. Here, indicate whether each material, system or method listed is relevant to your study. If you are not sure if a list item applies to your research, read the appropriate section before selecting a response.

Materials & experimental systems

n/a	Involved in the study	
☒	☐	Antibodies
☒	☐	Eukaryotic cell lines
☒	☐	Palaeontology and archaeology
☒	☐	Animals and other organisms
☒	☐	Human research participants
☒	☐	Clinical data
☒	☐	Dual use research of concern

Methods

n/a	Involved in the study	
☒	☐	ChIP-seq
☒	☐	Flow cytometry
☒	☐	MRI-based neuroimaging
