# Supplementary material for: Learning representations of chromatin contacts using a recurrent neural network identifies genomic drivers of conformation
Source: Nat Commun. 2022 Jun 28;13:3704. doi: 10.1038/s41467-022-31337-w (PMC9240038; doi:10.1038/s41467-022-31337-w)
Supplement: Supplementary file 4 — Description of Additional Supplementary Files [file 41467_2022_31337_MOESM4_ESM.pdf]

Title: Supplementary Data 1

Description: contains the ranking of transcription factor binding sites (TFBS) according to the aggregated feature importance scores.
